# Screening Ingredients from Herbs against Pregnane X Receptor in the Study of Inductive Herb-Drug Interactions: Combining Pharmacophore and Docking-Based Rank Aggregation

**DOI:** 10.1155/2015/657159

**Published:** 2015-08-03

**Authors:** Zhijie Cui, Hong Kang, Kailin Tang, Qi Liu, Zhiwei Cao, Ruixin Zhu

**Affiliations:** ^1^Department of Bioinformatics, Tongji University, Shanghai 200092, China; ^2^Shanghai Center for Bioinformation Technology, Shanghai, China; ^3^School of Pharmacy, Liaoning University of Traditional Chinese Medicine, Dalian, Liaoning, China

## Abstract

The issue of herb-drug interactions has been widely reported. Herbal ingredients can activate nuclear receptors and further induce the gene expression alteration of drug-metabolizing enzyme and/or transporter. Therefore, the herb-drug interaction will happen when the herbs and drugs are coadministered. This kind of interaction is called inductive herb-drug interactions. Pregnane X Receptor (PXR) and drug-metabolizing target genes are involved in most of inductive herb-drug interactions. To predict this kind of herb-drug interaction, the protocol could be simplified to only screen agonists of PXR from herbs because the relations of drugs with their metabolizing enzymes are well studied. Here, a combinational in silico strategy of pharmacophore modelling and docking-based rank aggregation (DRA) was employed to identify PXR's agonists. Firstly, 305 ingredients were screened out from 820 ingredients as candidate agonists of PXR with our pharmacophore model. Secondly, DRA was used to rerank the result of pharmacophore filtering. To validate our prediction, a curated herb-drug interaction database was built, which recorded 380 herb-drug interactions. Finally, among the top 10 herb ingredients from the ranking list, 6 ingredients were reported to involve in herb-drug interactions. The accuracy of our method is higher than other traditional methods. The strategy could be extended to studies on other inductive herb-drug interactions.

## 1. Background

In America, nearly forty percent of adults consume herbs or herbal products regularly every year and this number is still increasing [1]. One-sixth of them take herbal supplements together with prescribed drugs [2]. However, most of them do not realize that they are at the risk of potential adverse herb-drug interactions [3]. In order to avoid the medicine interactions as much as possible, it is urgent to discover the underlying herb-drug interactions.

**Figure 1:**
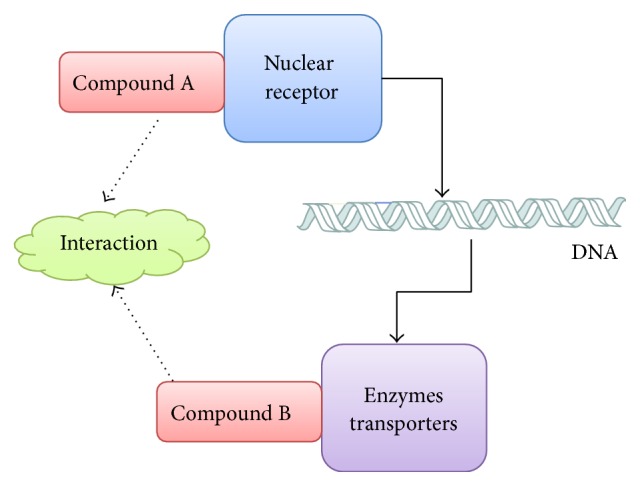
The mode of inductive drug interactions.

Herb-drug interactions, as well as drug-drug interactions (DDIs), are generally divided into two categories: pharmacodynamics (PD) interactions and pharmacokinetic (PK) interactions [4]. Many previous studies contributed to the explanation of molecular basis for drug interactions [5, 6]. In the late 1990s, it was found that ligand-activated nuclear receptors can regulate drug metabolism and transporter genes expression [7–9]. Nuclear receptors play an important role in the mechanism of PK interactions [10]. Based on that molecular mechanism (shown in Figure 1), herbal ingredients (agent A) can activate nuclear receptors and regulate metabolizing drugs (agent B) gene expression. Thus, the herbs could alter efficacy and toxicity of coadministered drugs. This process is called inductive herb-drug interaction [7, 11].

Pregnane X Receptor (PXR), as a member of nuclear receptor families, is involved in most of inductive herb-drug interactions through regulating drug-metabolizing gene expression [12, 13]. To predict the inductive drug interaction involving PXR, identifying ligands of PXR and drug-metabolizing enzyme/transporter could be done, respectively. However, because the relations of drugs and their metabolizing enzymes are well known, the key step of prediction would be simplified to only screen agonists of PXR. Several experimental systems* in vitro* have been developed for identifying agonist of nuclear receptors [14], such as cultured primary human hepatocytes and liver slices, humanized mouse models, transformed hepatocytes or cell lines, reporter gene assays, coactivator recruitment assays, and receptor binding assays [15–17]. But these experimental systems are low-efficiency and high-cost process to screen numerous molecules. Therefore, high-throughput and low-cost method for screening agonists of PXR is needed. Computational technique is just a good complementary for experimental systems.

In the past years, several computational methods have been used for virtual screening PXR's agonists, such as structure-based docking [18–20], ligand-based QSAR [21, 22], machine learning [18, 23], and pharmacophore model [24, 25]. Due to the large and flexible binding site of PXR [26], broad specificity of ligands, and the insufficient activity data [27], a comprehensive in silico strategy with both qualitative and quantitative analysis could be expected. The aim of our study is to propose a combined method of pharmacophore modelling, docking-based rank aggregation (DRA) for screening agonists of PXR. The method can provide aid for predicting inductive herb-drug interactions involving PXR. Also, it is applicable to predicting more herb-drug interactions involving other nuclear receptors.

## 2. Materials and Methods

### 2.1. Dataset

The complex crystal structure provides the binding information objectively, which is used for pharmacophore modelling and molecular docking. Three complex structures of PXR were obtained from the Protein Data Bank (PDB http://www.rcsb.org/pdb/home/home.do) [28], including 1NRL [29], 1ILH [26], and 3HVL [30].

266 compounds with EC_50_ values were obtained from the Binding Database (BindingDB http://www.bindingdb.org/bind/index.jsp) [31], which were selected as testing data for the pharmacophore modelling experiment. 71 compounds were labelled as active ligands (EC_50_ ≤ 10 *μ*M) and 195 compounds were labelled as inactive ligands (EC_50_ ≥ 10 *μ*M). In these 266 compounds, EC_50_ values of 107 compounds are numeric so that these compounds can be ranked by EC_50_ values (see Supplementary Table S1 in Supplementary Material available online at http://dx.doi.org/10.1155/2015/657159). The EC_50_-based ranking list (Rank_EC_) including 107 compounds was regarded as a reference list in the rank aggregation experiment.

In order to evaluate performance of our method, a dataset of herb-drug interactions was needed. 421 herbs were checked in the PubMed database by text mining method. 90 herbs were found to interact with 230 drugs forming 380 herb-drug interactions. Besides, molecular structures of herbal ingredients should be provided for pharmacophore modelling and molecular docking. Among 421 herbs, 820 ingredients structures were obtained from the PubChem database (http://pubchem.ncbi.nlm.nih.gov/).

### 2.2. Methods

#### 2.2.1. Pharmacophore Modelling

**Figure 2:**
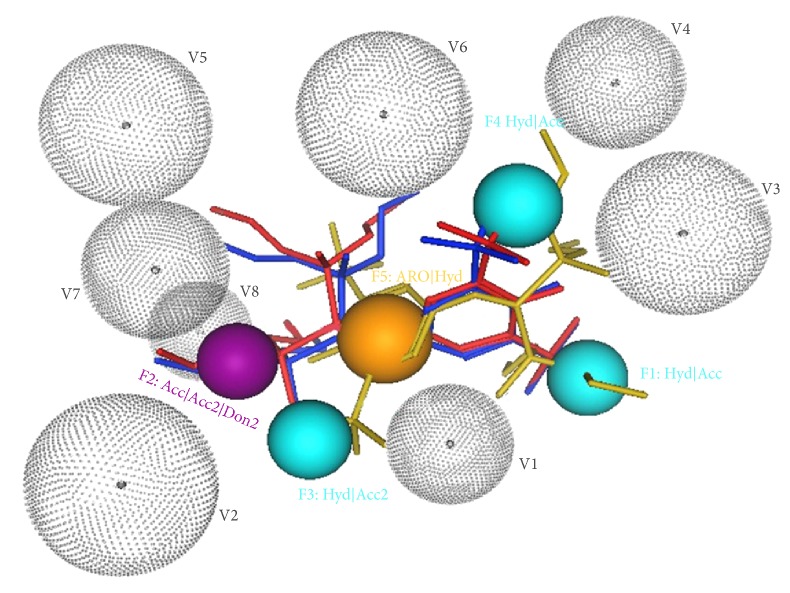
The pharmacophore of PXR (F1: Hyd|Acc; F2: Acc|Acc2|Don2; F3: Hyd|Acc2; F4: Hyd|Acc; F5: ARO|Hyd; V1–V8: excluded volume).

**Figure 3:**
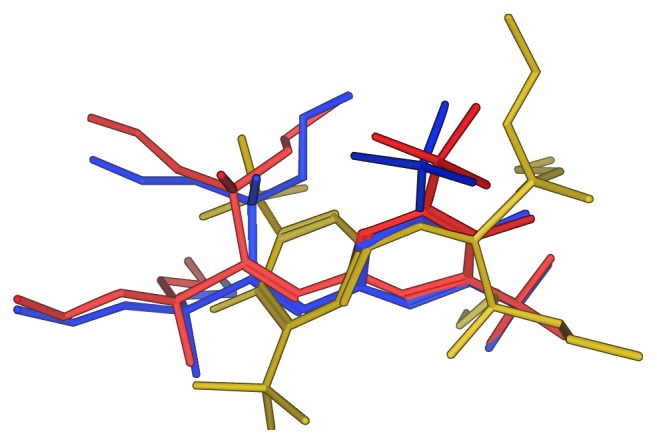
The molecular structure of template by superposing three SRL12813 in three different conformations.

As shown in Figure 3, three different conformations of SRL12813 were, respectively, extracted from complex crystal structures of PXR (PDB id: 1NRL [29], 1ILH [26], and 3HVL [30]). The red conformation of SRL12813 was extracted from complex 3HVL; the yellow one was extracted from complex 1ILH; the blue one was extracted from complex 1NRL. They were provided as template molecules. Pharmacophore was generated by collecting a common set of template molecules structural features. These structural features are related to the ligand's biological activity and recognition at binding site of receptor. In our model, five pharmacophoric structural features (shown in Figure 2) were fit by all template molecules. The process of pharmacophore modelling was performed in Molecular Operation Environment (MOE) 2008.10.

#### 2.2.2. Docking-Based Rank Aggregation (DRA)

Docking-based rank aggregation (DRA) is a two-step process. Firstly, candidate ligands filtered out by the former pharmacophore model were docked into PXR with four different energy scoring functions. The possibility of candidate ligands was ranked according their energy scores. Secondly, four different ranks from four scoring functions were aggregated to obtain a final rank.

The complex crystal structure of PXR (PDB id: 1ILH) was used to define the active site and dock with other molecules. Molecular docking was performed in MOE-Dock 2008.10. The way to place ligand was alpha sphere triangle matching with 4 different scoring functions (ASE Scoring, Affinity dG Scoring, Alpha HB Scoring, and London dG Scoring), respectively. The molecular mechanics force field was used to minimize energy of the system. 0.0001 kcal/(mol·Å) was chosen as the cutoff of the root-mean-squared gradient and maximum iterations was 1000 with their defaulted parameters. Finally, four ranked lists (Rank_AS_, Rank_AF_, Rank_AL_, and Rank_LO_) were calculated by 4 individual scoring functions.

Rank aggregation is a kind of multiview data analysis strategy aiming to fuse ranking results derived from individual views [32]. A final rank with views as comprehensive as possible, which is expected to better reflect the real rank, is worked out by aggregation of ranks from individual views [33].

Some concepts and details which are used in the process of rank aggregation are introduced below. Spearman's distance [34] is used to definition of distance between two given ranks:

(1) $$\begin{array}{c} S\left(L_{i},L_{j}\right)=\underset{t\in L_{i}\cup L_{j}}{\sum }\left|r^{L_{i}}\left(t\right)-r^{L_{j}}\left(t\right)\right|. \end{array}$$

Then, weighted Spearman's footrule distance between *L*_*i*_ and *L*_*j*_ is obtained via the following weighted summation representation:

(2) WSLi,Lj=∑t∈Li∪LjMrLit−MrLjt ×rLit−rLjt.

A function to detect the best list that is as close as possible to all the given ranks is defined as

(3) $$\begin{array}{c} \delta^{*}=\arg\min\emptyset \left(\delta \right),\\ \emptyset \left(\delta \right)=\overset{m}{\underset{i=1}{\sum }}w_{i}d\left(\delta ,L_{i}\right), \end{array}$$

where *w*_*i*_ refers to the list *L*_*i*_. *d* is Spearman's footrule distance between the best list *δ* ^*∗*^ and *L*_*i*_. The aim of rank aggregation is to discover the e distance between the best list *δ* ^*∗*^ and *L*_*i*_. The cross-entropy method was carried out to associate every two lists in our study [35].

To evaluate ranking performance in comparison with the control rank, discounted cumulative gain (DCG), a usual method to measure effectiveness of a web search engine algorithm, is used for evaluating performance of ranking. Two assumptions are acknowledged along with the use of DCG. One was that highly relevant items are more important when having higher ranks. The other is that highly relevant items are more important than irrelevant items. For a particular rank, the discounted cumulative gain accumulated position *p* was defined as

(4) $$\begin{array}{c} {DCG}_{p}={rel}_{1}+\overset{p}{\underset{i=2}{\sum }}\frac{{rel}_{i}}{{log}_{2}i}. \end{array}$$

The rel_*i*_ is the graded relevance of the result at the position *i*.

Due to the variety of lists in length relying on the query, the best rank would not be achieved if DCG is used along consistently. It was necessary for normalizing the cumulative gain of each rank. The normalized DCG (nDCG) was computed as

(5) $$\begin{array}{c} {nDCG}_{p}=\frac{{DCG}_{p}}{{IDCG}_{p}}. \end{array}$$

Finally, the average of every nDCG of lists is used to measure the similarity of two ranks. The range of nDCG is on the interval 0 to 1.

## 3. Results and Discussion

### 3.1. Pharmacophore Modelling

As a result of pharmacophore model, the true positive rate (sensitivity) is 53.52% (38/71) and the true negative rate (specificity) is 81.54% (159/195). The pharmacophore model of PXR is displayed in Figure 2. Remarkably, besides three different conformations of SRL12813, other agonists of PXR in the Protein Data Bank (PDB) were predicted exactly by our pharmacophore model such as RFP, HYF, PNU, and T0901317.

Two different views on how to superpose template molecules were used to construct the pharmacophore [36]. One is that the superposed conformation is gained by minimum energy [36, 37]. Yet, the other one is that the extracting conformations of ligand from its complex crystal structure are superposed directly [37, 38]. In our study, the latter method was adopted because its good performance was certified by the previous work [38].

### 3.2. Docking-Based Rank Aggregation (DRA)

Firstly, a list including 107 ligands of PXR was sorted by EC_50_ value and was regarded as reference list, named Rank_EC_. Secondly, 107 compounds were sorted again by calculated energy score from docking results. In molecular docking process, the binding energy score is used to evaluate binding affinity between protein receptors and ligands. It is estimated by individual scoring function. So four ranking lists of 107 compounds were generated depending on four individual scoring functions. As a result, nDCG values of these four lists were very low. It is indicated that these ranking lists from individual scoring functions were far from the reference list, Rank_EC_. In fact, the calculated energy score is weakly correlated to experimental binding affinity because individual scoring function just one-sided reflects the true binding situation. The low correlation was verified by previous studies [39, 40]. Our result is consistent with the viewpoint (shown in Table 1).

In order to find a ranking list of ligands, which was closer to the reference list, we aggregated ranking lists derived from docking results. The aggregated result showed that Rank_ABD_, which aggregated Rank_AS_, Rank_AF_, and Rank_LO_, is the best performance in all ranking lists. The nDCG of Rank_ABD_ is 0.7149, almost twice as high as any other lists (shown in Table 1). 107 compounds in every ranking list are shown in Supplementary Table S1.

Through our aggregated lists by docking result (shown in Table 2), two points are noteworthy. (1) The way to estimate the energy of hydrogen bond in Alpha HB Scoring (C) is much similar to that of Affinity dG Scoring (B), because such two scoring functions both are dependent on the favourable rule. In Affinity dG Scoring (B), two hydroxyl groups are assumed to interact in the most favourable way, but they are also discussed in Alpha HB Scoring (C), including non-sp3 donors and acceptors, sp3 donors and acceptors, and metals in the receptor. The potential redundant content rather than complementation between the two scoring functions caused that the nDCG of Rank_BC_ was lowest. (2) It was hypothesized that a large overlap of information occurs between Alpha HB Scoring (C) and ASE Scoring (A) on account that the same content on ligand atom-alpha sphere pairs was used to evaluate the energy. Likewise, the performance of Rank_AC_ is poor. Its nDCG is the third from bottom. So it was supposed that any good aggregated rank from the 4 scoring functions must not include Alpha HB Scoring (C) and Affinity dG Scoring (B) together, or Alpha HB Scoring (C) and ASE Scoring (A). Therefore, Rank_ABD_ is the best aggregated rank. It aggregates those views that are high complementary and and low redundant with each other. This viewpoint was coincident with the previous work [41].

### 3.3. The Prediction of Herb-Drug Interactions

The inductive herb-drug interactions were predicted through screening agonist of PXR from herbal ingredients. Every ingredient is contained by one or more herbs. An ingredient will be considered to have the potential of inducing herb-drug interaction if a herb, containing the ingredient, is reported in our herb-drug interaction database. The ingredient is regarded as the positive sample. The detection rate is used to measure the performance of the computational method, which is the ratio of positive samples in listed rank of screened ingredients.

305 ingredients were picked out from 820 ingredients of 421 herbs by our pharmacophore model. Then, three ranking lists of these 305 ingredients were generated, respectively, by molecular docking from three individual scoring functions (ASE, Affinity dG, London dG). A final list is obtained by aggregating these three lists. In the top 10 percent of the ranking list, the detection rate reached 0.6 (18/30). The whole results of rank aggregation are shown in Supplementary Table S2.

**Figure 4:**
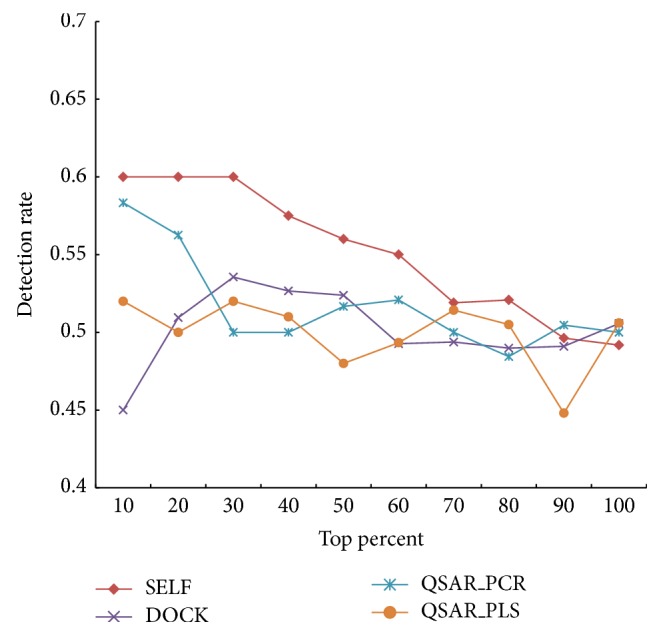
The detection rate in different ranking lists obtained by four methods.

As validity of methodology, the performance of our method was compared with traditional methods. We predict the inductive herb-drug interactions through screening agonist of PXR. Because candidate agonists screened by us are a ranking list, three methods for screening ligand of protein were chosen to compare, such as molecular docking, Partial Least Squares- (PLS-) based QSAR, Principal Component Regression- (PCR-) based QSAR. Likewise, 820 herbal ingredients are screened by different methods. As shown in Figure 4, the detection rate of our method (SELF) is higher than any other methods in different top percent of ranking. Our method indeed improves the performance of predicting herb-drug interactions. The result of ranking lists was shown in Supplementary Table S3.

As a part of screened result, the top 10 ingredients in final ranking list are shown in Table 3. They can be found in 14 herbs and 5 of these herbs were reported to be related to herb-drug interactions (shown in Table 3). Three cases are discussed in detail in the following.

**Case 1 (*Sophora flavescens*-theophylline interaction).**

Sophoraflavoside III and Sophoraflavoside IV are isolated from the roots of* Sophora flavescens* (SF), which is used to treat diseases such as diarrhea, gastrointestinal hemorrhage, and eczema [42]. Theophylline, also known as dimethylxanthine, is a methylxanthine drug for the treatment of respiratory diseases such as chronic obstructive pulmonary disease (COPD) and asthma [43]. The two herbal ingredients were potential agonists of PXR according to our pharmacophore and docking analysis. Theophylline is the substrate of CYP enzymes such as CYP2B [44, 45], CYP3A4, CYP1A2, CYP2E1, CYP1A1, CYP1B1, CYP2C8, CYP2C9, and CYP2D6. And the activated PXR by components of SF can induce the gene expression of these enzymes. Therefore, we predict that SF may change the metabolism of theophylline. The SF-theophylline interaction was evaluated in Ueng et al.'s experiment in 2010 [46]. They demonstrated that SF extracts reduced blood theophylline concentration via accelerating the clearance of theophylline in male Sprague-Dawley rats. Also, they were convinced that the expression of some enzymes metabolizing theophylline was upregulated such as CYP1A2, CYP2B1/2, and CYP3A4, all of which are target genes of PXR. The experimental results supported our predicted results. It is notable that our model not only predicted the SF-theophylline interaction successfully but also explained the potential molecular mechanism of interaction.

**Case 2 (*Sophora flavescens*-nifedipine interaction).**

This interaction was also observed in Ueng's study. Nifedipine is a dihydropyridine calcium channel blocker that primarily blocks L-type calcium channels [47]. A series of genes metabolizing nifedipine are regulated by PXR; SF extract could alter the metabolism of nifedipine by activating PXR. It was also found that the gene of CYP2C11 is upregulated by SF extract in Ueng's study and CYP2C11 is responsible for nifedipine oxidation [48]. But CYP2C11 is not target gene of PXR. So the explanation of SF-nifedipine interaction is outside scope of our model. Multiple interpretations for herb-drug interaction may exist simultaneously. On the one hand, several components in one herb hit multiple targets to influence drugs in different ways, like the influence on nifedipine by SF. On the other hand, different components in one herb sharing the same targets can act on the drug in the same way. Therefore, it is emphasized that our computational model only depends on the PXR-involving mechanistic mode and is incompetent to predict the PXR-independent interaction.

**Case 3 (Fritillaria-warfarin interaction).**

A clinical case of a 61-year-old man indicated that fritillaria lessens anticoagulation of warfarin [49]. The patient takes warfarin therapy regularly with a herbal product called Guilinggao resulting in his easy gum bleeding, epistaxis, and skin bruising. The main component of Guilinggao is fritillaria. Solanine which was one of fritillaria's ingredients was screened out as candidate agonist of PXR. Some of the enzymes which are related to warfarin's metabolism are modulated by PXR including CPY2C9, CYP1A2, CYP2C19, CYP3A4, and CYP2C8. According to our mechanistic mode for herb-drug interaction, fritillaria has an influence on warfarin's metabolism through changing the expression of some metabolic enzymes.

In our results, some ingredients were not reported to be associated with herb-drug interaction. Two potential interpretations are as follows: (1) the ingredient does interact with some drugs, but the interaction is not yet discovered* in vitro* and* in vivo*; (2) as a result of false positive from our pharmacophore model, the ingredients are not agonists of PXR.

## 4. Conclusions

In this study, a combinational in silico strategy was proposed to predict inductive herb-drug interactions. As a consequence, among 820 ingredients from 421 herbs, a ranking list of 305 ingredients was generated as candidate agonists of PXR. Among the top 10 herb ingredients from the ranking list, 6 ingredients were reported to involve herb-drug interactions. The strategy also could be extended to studies on other inductive herb-drug interactions. Besides, during the process of screening agonists for PXR, our pharmacophore model achieved a good performance across a broad dataset. What is more, the ranking result of traditional molecular docking was improved by rank aggregation. It is suggested that combining merits of scoring functions with less redundancies is a new orientation to optimize scoring functions.

## Supplementary Material

Three tables are in this Supplementary Material. Table S1 shows the ranking results for 107 agonists of PXR which is generated by different docking and rank aggregation methods. Table S2 shows the ranking results for 305 herbal ingredients of PXR which is generated by different docking and final rank aggregation methods. Table S3 shows Ranks for herbal ingredients, which are predicted as candidate agonist for PXR, were obtained by different methods.

## Figures and Tables

**Table 1:** The value of nDCG to measure distance between ranks.

Rank	nDCG
EC_50_	1
ABD	0.7149
AB	0.5397
D (London dG)	0.4599
ACD	0.4023
B (Affinity dG)	0.3972
BD	0.3961
AD	0.3947
BCD	0.3743
CD	0.3670
A (ASE)	0.3650
ABCD	0.3639
ABC	0.3609
AC	0.3423
C (Alpha HB)	0.3416
BC	0.3405

**Table 2:** The description of four scoring functions.

Index	Scoring function	Description
A	ASE Scoring	The distance between all ligand atom-receptor atom pairs and ligand atom-alpha sphere pairs.

B	Affinity dG Scoring	The enthalpic contribution to the free energy of various interaction including interactions between hydrogen bond donor-acceptor pairs, ionic interactions, metal ligation, hydrophobic interactions, interactions between hydrophobic and polar atoms, and interactions between any two atoms.

C	Alpha HB Scoring	Combination of two measurements between the geometric fit of the ligand to the binding site and hydrogen bonding effects.

D	London dG Scoring	The free energy for binding of ligand including the gain/loss of rotational and translational entropy, the loss of flexibility of the ligand, geometric imperfections of hydrogen bonds and metal ligation, and the desolvation energy of atom.

**Table 3:** The top 10 of final rank for candidate agonist of PXR from herbal ingredients.

Rank	Ingredients	Herbs	Reference (Y/N)
1	Sophoraflavoside IV	Sophorae flavescentis	Y
2	Hesperidin	*Sarothamnus scoparius *	Y
		*Scrophularia nodosa*; *Hyssopus officinalis*; *Tilia × europaea*;*Verbascum thapsus*; *Chlorella *	N
3	Sennoside C&D	*Cassia acutifolia *	N
4	Ginsenosides Rgl	*Astragalus membranaceus *	Y
5	Chlorophy II	*Medicago sativa*; *Urtica dioica *	N
6	Solanine	Fritillariae cirrhosae	Y
7	Senegenic acid	*Polygala senega *	N
8	Sophoraflavoside III	Sophorae flavescentis	Y
9	Phellanmurin	*Phellodendron amurense *	Y
10	Torulosic acid	*Juniperus communis *	N
