# Supplementary material for: Screening Ingredients from Herbs against Pregnane X Receptor in the Study of Inductive Herb-Drug Interactions: Combining Pharmacophore and Docking-Based Rank Aggregation
Source: Biomed Res Int. 2015 Aug 3;2015:657159. doi: 10.1155/2015/657159 (PMC4538340; doi:10.1155/2015/657159)
Supplement: Supplementary file 1 — Three tables are in this Supplementary Material. Table S1 shows the ranking results for 107 agonists of PXR which is generated by different docking and rank aggregation methods. Table S2 shows the ranking results for 305 herbal ingredients of PXR which is generated by different docking and final rank aggregation methods. Table S3 shows Ranks for herbal ingredients, which are predicted as candidate agonist for PXR, were obtained by different methods. [file 657159.f1.pdf]

Supplementary Materials

Table S1: Ranks for 107 agonists of PXR from EC_{50}, docking and rank aggregation.

107 compounds are labeled by monomer identifier in the Binding Database.

Rank	EC_{50}	ASE(A)	Affinitiy dG(B)	Alpha HB(C)	London dG(D)	AB	AC	AD	BC	BD	CD	ABC	ABD	ACD	ABCD
1	21670	50347619	50323533	50347619	50347619	50323533	50261050	75527	50333724	50333724	50333724	50347619	21670	50347619	50347619
2	50251088	50333723	50333724	50323530	50333724	50333724	50333711	50347619	50323530	50323533	50323530	50316513	50333724	50333724	50316513
3	50251070	50333716	50323530	50333724	50323533	21670	50333724	50323533	50323533	50347619	50323533	50333724	50261010	50333719	50333724
4	21673	50333711	21672	50323533	75527	50333722	50323530	50316513	50261010	50316513	50347619	50323530	21673	50333723	50323530
5	19993	50333724	50260967	21672	21670	50261050	50347619	50333723	50260967	50261010	50261050	50323533	50323530	50333716	50323533
6	50251092	50333713	21673	69532	21673	50347619	50316513	50333724	21672	75527	21672	50260911	50333713	50261010	50260911
7	21671	50333722	50260911	50260966	21666	50333719	50333713	21670	50260911	21673	21666	50261010	50347619	50333711	50261010
8	50251081	50333719	21671	21666	50181559	50316513	50333719	50261050	50340800	21672	69532	50260967	50260966	21670	50260967
9	50251048	50348294	50316513	50251088	50323530	21673	50323533	50333713	21673	21671	21671	50333713	50323533	21666	50333713
10	50251056	50261050	50261010	50340800	50251110	21671	50340800	50261008	50347619	50323530	19993	50251092	50333722	50261050	50251092
11	50251069	21670	50340798	50261050	50333723	50261010	50333723	50333719	50333719	21670	21673	50333711	50260911	50181559	50333711
12	50251089	50323533	21670	50333716	19993	50333716	50333716	50333711	50333716	21664	50251088	21666	50333716	21672	21666
13	50251068	50348308	50252940	21673	21672	50323530	21666	50333722	21671	50260967	75527	21671	50333719	50323530	21671
14	50251102	50323530	50260880	21664	21663	50251088	50261010	50260911	50181559	50340829	50252940	50251088	50316513	21673	50251088
15	50251090	50348296	50251067	50260911	50316513	21672	50348294	50260966	50251088	50340798	50181559	50261050	75527	75527	50261050

16	50251091	50261010	50251110	50251067	75532	50333723	50251110	50333716	50251110	50260911	50260911	50348308	50260967	50333713	50348308
17	50251110	50316513	50333719	50261010	50261008	50260911	21665	50348296	50251057	50260966	50251110	50333716	50251092	50323533	50333716
18	50251099	50251092	50251079	50333711	21671	50261008	21670	50252940	50251109	50251088	50251050	50333722	50251088	50252940	50333722
19	21665	50260966	50261008	50251057	50252940	50260967	50260967	50340800	50261050	19993	50260967	50340800	50340820	50260966	50340800
20	50348293	50340800	19993	50251048	50261010	50333711	50251092	50251088	50251049	50251079	50261008	50251090	21671	50251088	50251090
21	50348292	50251090	50347619	50181559	21664	21666	75527	50251110	50261008	50261008	21665	21670	50252940	21665	21670
22	50348294	50260911	50260966	50251050	50261050	50251110	50251088	50323530	50260908	21666	50316513	50260966	50181559	21671	50260966
23	50348308	50348293	50251088	50260967	50340829	50348294	50261008	50348294	50260880	50261050	75488	50348294	50251067	50251070	50348294
24	50348291	75527	50333716	50316513	50260911	50251092	21671	50260910	50251050	50252940	21663	21673	50260880	50251110	21673
25	50348296	50348292	50333722	50333723	21665	50260797	50252940	21672	50251058	50181559	50251090	50340798	50251048	50251048	50340798
26	21666	50348291	50260797	21663	21668	50348308	50260910	50181559	50251078	21665	50251079	50251101	50348293	75532	50251101
27	50251050	50251110	75527	21671	21667	50348292	50251101	19993	50340820	50340794	50333723	50348293	50348294	50251090	50348293
28	50251059	50261008	50251102	50333719	50333719	50251067	50251079	50251102	50260938	21667	50260908	50181559	50340800	50261008	50181559
29	50251079	50251088	50348294	50251090	50251088	50251102	50260966	50251049	50333722	50333716	21670	50251050	50333723	50260908	50251050
30	50252940	21666	50260910	75532	50251050	50251069	75488	50260938	21664	50251049	50251092	50260908	50340829	50260911	50260908
31	50251100	50251089	21665	50251110	50333716	21665	50260911	50251091	50251090	50333722	50260966	50333719	21666	50348292	50333719
32	50251101	50260910	50340820	75488	50251048	50251109	50251048	21665	19993	21663	50251067	50251051	50333711	50260967	50251051
33	50251058	50260967	50251089	50251092	50340802	50260910	50251051	21666	50340798	50348296	50333711	50251079	50261050	50316513	50251079
34	50251057	50260880	50340800	50348294	50251070	50260908	50251049	50261010	50316513	50251089	21664	50252940	50348308	50251089	50252940
35	50251109	50251051	50251092	50333713	50340794	19993	50251109	50251070	50260910	50333713	50251102	50333723	50251089	21663	50333723
36	50251067	50260936	50251049	75527	50260967	50348293	50181559	21673	50333723	50251110	21668	21663	50340794	50340802	21663
37	50251051	50260797	50333713	50251079	50251092	50333713	50251057	75488	50251048	50251100	50260910	21672	50251068	75450	21672
38	21664	50251070	50348308	50252940	50251067	50251051	21663	21671	75532	50348294	50340800	50251048	21663	50251092	50251048
39	50251078	50251091	50251050	50251101	50260966	50181559	21672	50251090	50251067	50251056	50251101	21665	21664	50348294	21665
40	50333711	50251049	50261050	50251109	50333711	50340798	50251067	50260936	21666	50260880	32292	50251049	50251070	50251078	50251049
41	50333713	50251079	50251059	50260938	50251079	50260880	50348308	75532	50348294	50251050	50251051	50251081	50340798	50251099	50251081

42	50261010	50340820	50340794	50261008	50333713	50340794	50260797	50348308	50333711	50260797	75532	21664	50261008	21668	21664
43	50333716	50181559	50181559	19993	50251102	50340829	21664	50340794	50260966	50251048	50261010	50260880	50251060	21664	50260880
44	50251049	50340802	50251109	21670	50251109	50251090	75450	50251079	21670	50260938	50260936	19993	50251079	50340820	19993
45	50333719	50340798	50251051	50251060	50260908	50251091	50251050	50251067	21663	50251070	50333716	50251089	50251109	50333722	50251089
46	21672	21671	50340829	50251078	50348294	50260938	75532	21664	50333713	50251057	50251109	50261008	50260938	50340800	50261008
47	21663	21673	50251100	50260910	50251099	50340820	75525	50251092	50348296	75475	50251089	75488	50348291	50251102	75488
48	50260966	50260908	21666	50251049	50251057	50251100	50348296	50340829	50251092	50340820	50251059	50251067	50348296	50251051	50251067
49	50251060	50252940	50251090	50251058	75505	21668	50333722	50251051	75527	50251059	50251069	69532	50251100	50260797	69532
50	50239407	50251067	50251048	50333722	50251100	50252940	50251059	50260797	50251100	50251051	75505	50251057	50251069	71633	50251057
51	50260880	21665	50251070	21665	50251068	50251079	50251090	21663	50348293	50333719	50251048	50260797	50251059	50251057	50260797
52	50260911	50260938	21664	21668	50251090	50251089	50260938	50260880	50340829	50348308	50340829	21667	75475	50251059	21667
53	50260797	50251048	50251078	50260908	50260880	50251050	75505	50251109	50251102	50348293	50333719	50251058	50251090	69532	50251058
54	50333722	50251057	50348296	50251051	50251049	50260966	50317579	75469	32292	50333723	75517	50260910	19993	50251109	50260910
55	50260936	50340794	50260938	50251059	50317579	50251070	50251102	75525	50251060	50251102	50260797	50348296	50348292	19993	50348296
56	50340798	50251069	21663	75475	75488	21669	50251070	50251056	21667	50251090	50251091	50251110	50340802	75525	50251110
57	50260967	75532	50251101	50260936	75481	50251059	21668	50260908	50251081	50260936	50251057	50251069	21672	50251091	50251069
58	50317579	75488	50251069	50340820	75475	50251048	50340820	50251050	75479	50251078	50340798	75475	50251051	50251101	75475
59	50260908	50251068	50251057	50251081	50333722	50348296	21673	50251068	48711	50251109	50348294	50251078	50251057	50251081	50251078
60	50340800	50251102	50333723	75505	50260910	50251060	50340829	50251048	50251079	50251091	50251049	50340820	50260797	50251060	50340820
61	50340802	75525	50260908	50251089	50251051	50348291	50340798	50251081	50251089	50260908	21667	50251100	50251110	50251056	50251100
62	50316513	50251059	50348293	50340829	50260938	50251057	50251056	50348292	50251059	50251060	75491	50340802	50251102	50260910	50340802
63	50347619	21672	50317579	50340802	21669	75525	50251078	50348291	50348291	75491	50340820	75527	75532	75488	75527
64	50181559	75450	50348291	50260880	50348296	50251058	50348292	75481	50260797	50251099	50260938	75532	50260910	50251079	75532
65	50333723	50251109	75479	50251102	69532	75532	50251100	21668	50251068	50340800	50251078	48711	50260936	50340798	48711
66	50260910	50251056	32292	50251100	50260936	50251101	75475	75475	50251101	75488	50333722	50251070	50317579	50251050	50251070
67	50261008	19993	50333711	21667	50251091	75488	50260880	75486	75488	50251068	75481	50340794	32292	50260938	50340794

68	50340794	50239407	75488	32292	32292	75491	50260908	50348293	50348308	75479	50251068	75517	50251081	50251067	75517
69	21669	71633	50348292	50251099	50340800	75527	50260936	50340802	75475	21669	50333713	50348292	50251058	75491	50348292
70	21668	50251081	50251060	48711	50260797	50251049	50251089	75491	50348292	50251067	50251100	75450	50260908	50348308	75450
71	21667	21664	75475	75479	50340820	50317579	50251069	50260967	50251056	50340802	71633	50251060	75488	21667	50251060
72	50333724	50251101	75532	50251070	50251089	50340800	75486	21667	21665	50251069	50251081	50260936	50251050	50260880	50260936
73	50260938	75469	50251056	75481	50340798	50260936	50251081	50340798	69532	50333711	50251058	75449	48711	32292	75449
74	75488	75459	50251058	50251056	50251078	75459	19993	50239407	50252940	75505	50251056	50251056	71633	50260936	50251056
75	69532	50251050	50340802	75459	50251101	75475	75459	50251100	50251070	50260910	75475	50251068	50251091	50251069	50251068
76	50340829	50340829	50251099	50348293	50251056	75479	50251060	50251059	50251099	75477	50260880	50251059	75450	50340794	50251059
77	50340820	50251058	50251091	75486	50251069	50251068	48711	50251099	50317579	48711	50251070	75469	75449	50251100	75469
78	50261050	50251099	50251068	50260797	75477	50239407	50340802	50251089	50251091	50251081	50317579	50251102	21665	50340829	50251102
79	75450	75491	75491	50348296	50251059	21664	71633	69532	21668	21668	21669	50251109	50251049	50348296	50251109
80	75491	75475	50251081	50340798	75491	50251056	75469	50251058	71633	50348292	75486	50260938	75492	75517	50260938
81	75525	50251060	21667	50251069	50251081	50251099	50251091	50251057	50251051	75492	50348291	75479	75479	50348293	75479
82	75517	50251078	21668	50348308	50251060	21663	50348293	75517	50251069	50251101	50340794	50348291	50251101	50348291	50348291
83	75534	50251100	75487	75510	71633	75469	50348291	50317579	75481	50251058	50340802	21668	75505	50251049	21668
84	48711	50317579	50260936	50251068	75486	75450	75492	50251101	75492	32292	50348308	50340829	50251078	50239407	50340829
85	75510	75481	75469	75450	75487	50251078	69532	75449	50340794	75532	50251060	21669	75459	21669	21669
86	75492	21668	21669	21669	75479	75505	50251068	50340820	21669	50251092	50348293	32292	50251056	50251068	32292
87	75481	21663	48711	50348292	50348291	75486	50251058	75505	75491	71633	75477	50239407	50251099	75479	50239407
88	75479	75447	71633	50251091	50348308	50340802	21669	50251060	50239407	75447	48711	75481	21667	75505	75481
89	71633	48711	75520	75517	75449	48711	75481	50251069	50260936	75481	75487	75525	50239407	75481	75525
90	75520	75479	50239407	50348291	48711	75447	50340794	50251078	75505	50239407	75450	50251099	75491	50251058	50251099
91	50264073	21669	75450	75525	75492	50251081	75447	71633	75486	75459	75525	71633	75469	48711	71633
92	69214	75505	75492	75491	50239407	32292	32292	75447	75487	75450	50348296	75459	75481	50317579	75459
93	75532	75449	75505	75476	75447	75487	75479	75450	50340802	50348291	75479	50251091	69532	75486	50251091

94	75486	75517	69532	75492	50348292	69214	50239407	75459	75477	50317579	50348292	50317579	21669	75475	50317579
95	75469	32292	75525	75447	50251058	50264073	50251099	69214	75525	75449	50251099	75492	21668	75447	75492
96	75449	75486	75449	75477	75450	71633	75534	75492	50264073	69532	75447	75491	75525	75510	75491
97	75477	21667	75459	75449	75517	75520	75517	32292	75520	75487	75459	75520	75447	75459	75520
98	75459	50264073	75486	50317579	50348293	75481	75491	75479	75469	69214	75510	75505	75486	75492	75505
99	32292	69532	50264073	71633	75510	21667	75449	21669	75459	75525	69214	75510	75520	75449	75510
100	75527	75487	75477	50340794	69214	69532	50264073	75477	75450	75486	75534	69214	75487	75476	69214
101	75476	75492	75481	50239407	75469	75492	21667	48711	75517	75517	75449	75487	50264073	75487	75487
102	75475	75520	75517	75469	75525	75510	75476	75487	75510	75469	75492	75486	75517	75520	75486
103	75505	75477	69214	75534	75459	75517	75510	75534	75534	75520	75476	75447	75510	75469	75447
104	75487	69214	75447	69214	75476	75449	75477	75520	75449	50264073	50239407	75477	75477	75477	75477
105	75447	75476	75510	75487	75534	75477	75520	75510	75476	75476	75520	50264073	69214	75534	50264073
106	50323530	75510	75534	50264073	50264073	75534	75487	50264073	75447	75510	75469	75476	75476	69214	75476
107	50323533	75534	75476	75520	75520	75476	69214	75476	69214	75534	50264073	75534	75534	50264073	75534

Table S2: Ranks for 305 herbal ingredients predicted as candidate agonist of PXR from docking and rank aggregation.

305 ingredients are labeled by PubChem Compound Identifier.

The final rank is obtained by aggregating three ranking lists (ASE, Affinitiy dG, Alpha HB).

Rank	ASE(A)	Affinitiy dG(B)	Alpha HB(C)	London dG(D)	Final
1	432524	6477652	54605677	432524	3083427
2	3083427	6441188	108062	108062	10621
3	108062	6433489	6441188	5281233	161940
4	6433489	54605677	5199	3083427	432116
5	197563	5281233	161940	250395	6477652
6	54605677	108062	197561	161940	262500
7	262500	125210	432524	6433489	200662
8	197561	6441498	3083427	197561	197563
9	6477652	5199	44257279	441877	193876
10	18772499	161940	6433489	54605677	12314445
11	197977	275182	24847857	44257279	6433489
12	432116	115149	250395	197977	119245
13	11953919	12314445	5320686	5199	432524
14	24847857	86821	168849	197563	108062
15	119245	432116	197977	6441188	197561
16	125210	5271805	5280805	432116	5315459
17	128327	5320686	6475724	6477652	44257279
18	441877	200662	5271805	3084742	5281233
19	276547	16061280	432116	6441498	6441498
20	161940	5351212	16211077	24847857	5280746
21	441298	11953919	5281800	11953919	6441188

22	200662	10621	10621	5315459	11953919
23	12314445	5281600	193876	3082301	441381
24	5199	276547	156073	10621	5199
25	5281233	5315459	441381	119245	5271805
26	86821	5281793	6441498	5281793	180948
27	275182	441298	174157	125210	125210
28	161388	5281613	5280746	5281751	24847857
29	94475	44257675	5315459	156073	480768
30	168849	54611038	115149	5281613	49775362
31	5351212	442664	442664	5280805	275182
32	2116	156073	5281613	262500	5281613
33	44257279	441381	44257675	5281600	5281051
34	442195	2116	3082301	442664	174157
35	14986	5281051	441877	44257675	17473
36	462196	6453770	6477652	193876	435402
37	65033	9954815	262500	54676854	441877
38	10621	180948	5281600	5281800	462196
39	115149	5281751	185617	5281051	155256
40	3082301	432524	17473	107905	5281800
41	5281800	128327	5281807	5320686	54605677
42	10219	5318893	64982	49775362	5281852
43	5280805	14986	5320844	65064	381851
44	6441188	94475	5281542	462196	22524411
45	442217	5318891	462196	168849	44259516
46	5281613	6475724	197563	5281777	5351212
47	442664	10389806	119245	5271805	72301

48	49775362	5317419	10389806	16211077	442195
49	5281542	3083427	275182	441764	31553
50	5462438	65033	54676854	5280459	5318891
51	180948	441764	5280633	3033957	197977
52	435402	250395	6453770	5281675	5280805
53	5320686	11982640	3033957	5280746	54611038
54	193876	5318880	5280804	180948	5281542
55	156073	3081405	5281793	128327	6442181
56	5318880	10219	162350	5281673	46173967
57	441381	5281542	5280459	17473	11982640
58	44563198	193876	16211710	185617	5462438
59	65071	442217	5318891	174157	442664
60	5318893	10455036	180948	5351212	12305761
61	68051	5281800	5281751	65084	5280804
62	5318891	435402	5351212	44257224	5318880
63	5280746	44563159	441764	5281643	107905
64	54611038	462196	5383409	5280804	5281807
65	44257675	5462438	6037	5281603	23915
66	16061280	16211710	5281777	44259516	5281751
67	9954815	44563198	5281233	5281792	5281600
68	5839585	441663	22524411	5481237	276547
69	6442181	5280805	5281643	10389806	5320844
70	13872451	442195	503737	115149	168849
71	6441404	65071	44257224	49770697	16211710
72	196699	5481237	5281673	5281807	168114
73	155256	44257279	164146	31553	6475724

74	5281753	31553	49775362	22524411	10219
75	6475724	5282102	25202038	3085830	5318893
76	72625	381851	22524410	441381	5281753
77	44257224	161388	52940094	5282102	117440
78	358902	13872451	5318880	5320844	44563198
79	6441405	9979767	12305761	5490064	10455036
80	11982640	168849	117440	64982	5481237
81	381851	181796	46173850	44260098	34059
82	5281852	44259516	5480505	5281542	1548943
83	5281754	49775362	94475	16211710	181796
84	6453770	5280933	107905	5318891	115149
85	23915	5383409	44258916	5318880	64982
86	54600668	44563121	5464155	5281775	441764
87	73114	65064	5317419	5280633	73466
88	168115	5281377	44259516	6475724	5280441
89	5271805	24847857	5481237	6453770	6419993
90	44259516	68051	161388	5383409	493570
91	99091	441877	5490064	5480505	168115
92	3084742	480768	5489486	162350	94475
93	443421	969516	31553	5281377	65064
94	5315459	5280746	5281051	189065	3082301
95	10455036	168115	9954815	114776	16211077
96	44563159	197561	114776	25202038	9954815
97	5281777	442647	276547	5281654	503737
98	3033957	185617	49770697	275182	261859
99	261859	114776	10219	42607998	44563159

100	6758	493570	12314445	11982640	73467
101	356660	3033957	441663	276547	128327
102	9979767	54600668	44563159	94475	6453770
103	5281125	44257224	5318893	10219	5320686
104	22524411	6442181	5281377	12305761	98369
105	6167	5281852	5282102	44258916	250395
106	44257399	44260098	442195	11948668	5317419
107	31553	5281777	5281675	5280441	442101
108	181796	503737	5281792	200662	5281673
109	44563121	5280804	9979767	181796	44258916
110	16211710	5281867	73466	480768	442435
111	174157	5280450	12315119	164146	196699
112	5480505	42607998	42608131	442662	5315263
113	6438142	5281792	5462438	72277	358902
114	168114	46173967	92735	44563198	443421
115	17473	92885	42607998	162464	5281867
116	46173966	92735	5281417	12314445	189065
117	643684	5281807	5839585	5317419	22524410
118	46173967	643684	65064	22524410	356660
119	72616	5281775	128327	65071	3085830
120	480768	5281406	5280441	16061280	5281794
121	441663	72625	3085830	5464461	72277
122	3085830	44135670	443354	10455036	366269
123	163776	5281643	107971	435402	49770696
124	117440	5280459	261859	54600668	25202038
125	445639	107971	44563198	5322079	65033

126	52940094	5280441	93045	73467	5383409
127	12315119	155256	442217	381851	5318998
128	46173965	22524411	200662	107848	12315119
129	72301	3082301	11948668	73466	54600668
130	10389806	107905	13872451	513197	86821
131	1309	16211077	10455036	5464155	5281775
132	34059	11005	5322079	5281608	5281777
133	6441498	34059	441298	13872451	440936
134	5281807	445639	381851	6037	9276
135	5481237	12305761	125210	443354	65071
136	92885	22524410	11982640	44563159	44257224
137	5281793	363452	5320351	107971	164146
138	5281752	3446460	99091	12309507	5282102
139	5318998	6442675	969516	42608131	5464461
140	164146	5281614	65033	392443	441298
141	6419993	49770697	44563121	34059	445070
142	5282102	5281125	1309	599030	5281793
143	442643	513197	5281603	3081405	5489486
144	5320351	22179	5464461	93045	3033957
145	54676854	358902	10154	261859	68051
146	44260098	16573	88708	46173850	5281417
147	5280450	64982	435402	441298	11948668
148	441764	25202038	442435	442514	668081
149	22524410	167718	366269	99091	5490064
150	42607998	5464155	34059	12315119	44563121
151	1548943	5281675	11953919	168114	37834

152	503737	11442	392443	9954815	72616
153	5280804	42608131	440936	44563121	442810
154	5281600	442810	181796	161388	91520
155	64982	68081	442101	2116	443322
156	167718	10154	107848	442195	2116
157	24197663	73114	12309507	5320462	513197
158	609998	174157	44260098	54611038	5464155
159	158940	46173966	5281608	5318893	442662
160	73467	6758	68051	5280666	11005
161	44258916	5322079	65084	441663	162350
162	185605	5281673	5280699	100058	9979767
163	11442	46173965	5281406	442435	46173966
164	185617	5490064	100058	5280699	443354
165	3081405	5280633	158940	49770696	44257675
166	5281751	5318624	2116	442101	158940
167	250395	443421	54611038	5315263	46173850
168	3661	443322	5315263	503737	98570
169	12305761	5280699	6850754	65033	5322079
170	5280933	117440	161036	358902	5281377
171	5281794	168114	14986	117440	107971
172	162350	6441405	54600668	9979767	5280933
173	3082205	10233	6441405	5281406	161388
174	5320844	1309	442647	356660	445639
175	5383409	163776	73467	37834	5280699
176	5281643	5281753	480768	14986	442514
177	5281673	5318998	163776	6441405	100058

178	162464	158940	5280666	158940	5280450
179	42608131	44258916	72625	98369	5280459
180	443322	37834	73160	443322	5280633
181	442435	6419993	23915	91520	46173965
182	5490064	54676854	167718	5281417	363452
183	366269	442435	72616	5318624	442217
184	442647	162350	493570	440936	6758
185	100058	442514	46173967	493570	10389806
186	25202038	5281754	91520	282014	6441404
187	5281131	164146	185605	52940094	73114
188	108053	99091	5281654	5281867	72625
189	11005	107848	5318624	5489486	42607998
190	107971	366269	65071	5281754	6850754
191	442793	196699	599030	23915	162464
192	443354	3084742	358902	442217	5839585
193	11948668	98369	189065	73160	599030
194	6857681	5646	442810	161871	44260098
195	92735	5280666	162464	442810	92138
196	107848	261859	168114	185605	16061280
197	114776	24197663	443322	5320351	442793
198	24832101	442101	5281855	163776	185617
199	5281377	7605278	91458	120693	609998
200	6857559	5480505	3084742	5281614	5318624
201	5280441	5315263	6758	442793	5281131
202	34458	4970	5320462	24752823	68081
203	442810	5489486	356660	88708	5480505

204	44135670	356660	16573	5318998	91458
205	5281775	392443	513197	5280450	5281125
206	5315263	5318869	37834	6419993	99091
207	98570	72301	3446460	5462438	44257399
208	114843	189065	5281775	92735	6438142
209	5280459	24832101	5281614	5281855	5281643
210	37834	5320844	86821	5839585	42608131
211	5281608	161036	5318998	7098673	156073
212	10143	12315119	177023	167718	5320462
213	5281675	98570	168115	1548943	177023
214	4970	5839585	6441404	91458	8193
215	6850754	5281131	5281754	6442675	392443
216	599030	73160	363452	5318869	167718
217	65064	609998	6167	86821	5320351
218	5281051	46173850	72301	10233	93045
219	3446460	23915	155256	576072	24197663
220	91520	6857559	5281753	72301	52940094
221	91458	5281417	668081	366269	5281603
222	93045	52940094	442514	155256	44135670
223	98369	445070	72277	161036	14986
224	161036	668081	16061280	168115	65084
225	16573	1548943	5646	6442181	126543
226	969516	443354	5318869	16573	442266
227	88708	124966	932	5281753	5281792
228	668081	73466	7098673	73114	5281654
229	8193	5281794	196699	6167	120693

230	168136	12309507	44257399	10143	92735
231	10154	6441404	609998	72625	5281406
232	10233	126543	439503	34458	3081405
233	392443	282014	92885	6850754	73160
234	101300	44257399	161871	5281852	107848
235	46173850	3085830	442662	124966	3084742
236	5281867	8193	282014	177023	5281608
237	442662	932	576072	1309	6441405
238	92138	442793	442266	969516	24832101
239	73466	5281654	44135670	24197663	6167
240	493570	5281752	46173966	46173967	163776
241	445070	5281855	9276	46173966	5281754
242	513197	442643	22179	5984	10233
243	5280699	34458	6442675	72616	12309507
244	117301	5320462	6419993	5646	442643
245	5280633	92138	24752823	9276	54676854
246	107905	5281608	1548943	5281794	11442
247	5320462	49770696	443421	3446460	5281675
248	9276	92265	5281131	5280933	114776
249	107938	72616	5281867	10154	5281855
250	6037	72277	98369	22179	441663
251	189065	5281603	98570	108053	3446460
252	5317419	6857681	442793	5281752	4970
253	5464461	5320351	10143	4970	24752823
254	124966	177023	98455	68051	88708
255	363452	108053	10233	443421	107938

256	5318624	519662	5281752	44257399	18772499
257	5322079	185605	46173965	196699	49770697
258	5281417	9276	643684	3082205	3082205
259	519662	162464	445639	6441404	643684
260	5281792	100058	5281852	6758	5646
261	16211077	93045	120693	363452	5318869
262	442101	599030	4970	439503	161036
263	22179	262500	6438142	98570	10154
264	92265	439503	101300	107938	6857681
265	229851	6438142	34458	442266	92885
266	5281406	65084	11442	445639	969516
267	5464155	117301	3081405	609998	185605
268	177023	88708	49770696	643684	7098673
269	7605278	3661	442643	5281125	7605278
270	576072	98455	107938	6438142	1309
271	5281603	442662	108053	101300	442647
272	5646	91458	445070	442643	13872451
273	98455	5464461	24197663	932	114843
274	49770697	10143	124966	92265	10143
275	5318869	197977	68081	114843	6442675
276	442266	73467	3661	92885	5280666
277	5280666	6167	6442181	11005	6037
278	6442675	101300	3082205	229851	282014
279	439503	11948668	5280933	668081	576072
280	440936	168136	5280450	11442	5281614
281	442514	6037	5281125	8193	5281752

282	5489486	107938	5281794	6760	168136
283	7098673	91520	6857681	6613	108053
284	131592	576072	6760	44135670	131594
285	131594	17473	10659	117301	3661
286	73160	442266	114843	445070	519662
287	24752823	3082205	126543	46173965	6857559
288	6760	440936	73114	5281131	5984
289	126543	120693	11005	92138	101300
290	68081	114843	131594	98455	16573
291	65084	119245	6613	6857681	161871
292	932	197563	229851	10659	439503
293	5281654	7098673	7605278	7605278	22179
294	72277	6760	519662	442647	124966
295	10302	131592	5984	131594	229851
296	6613	229851	92265	68081	34458
297	282014	161871	168136	3661	92265
298	12309507	24752823	117301	6857559	932
299	5281614	10659	6857559	126543	117301
300	120693	6613	24832101	519662	131592
301	161871	10302	131592	24832101	6760
302	10659	131594	8193	131592	10659
303	49770696	6850754	10302	168136	6613
304	5281855	5984	92138	10302	98455
305	5984	18772499	18772499	18772499	10302

Table S3: Ranks for herbal ingredients predicted as candidate agonist for PXR by different methods.

rank	Docking	QSAR_PCR	QSAR_PLS	SELF
1	45480624	18772499	18772499	3083427
2	16129778	6477652	6477652	10621
3	441877	24434	5281520	161940
4	5199	5281520	5373727	432116
5	161940	6433489	12409	6477652
6	197563	5373727	2116	262500
7	3083427	54605677	14986	200662
8	128229	5281852	54605677	197563
9	432524	2116	5281852	193876
10	441895	14986	24434	12314445
11	250395	12409	167551	6433489
12	122097	167551	68406	119245
13	197977	5281858	5281858	432524
14	24847857	68406	6433489	108062
15	198016	8222	89841	197561
16	432116	5281515	5281515	5315459
17	108062	5322111	5322111	44257279
18	196359	12401	8222	5281233
19	197561	89841	65078	6441498
20	3084742	11635	12401	5280746
21	44257279	65078	168115	6441188
22	65064	12398	11635	11953919
23	6441188	11006	12398	441381
24	125210	12391	3082205	5199

25	5281793	3082205	24832101	5271805
26	14982	5280489	636458	180948
27	262500	636458	11006	125210
28	5280805	168115	441298	24847857
29	11953919	24832101	12391	480768
30	168849	6506548	6506548	49775362
31	5281792	8141	52948006	275182
32	442664	52948006	54600668	5281613
33	107905	14350	14350	5281051
34	441381	5281235	5280489	174157
35	5281600	54600668	13584	17473
36	441764	24502	10104370	435402
37	5281613	441298	521253	441877
38	11953920	8193	168114	462196
39	174157	10104370	11442	155256
40	5281800	521253	526503	5281800
41	5281751	13584	8141	54605677
42	5281051	8175	5280795	5281852
43	5271805	526503	8193	381851
44	6475724	31289	6473881	22524411
45	5315459	8914	5281428	44259516
46	156073	454	44135670	5351212
47	6441498	168114	5281235	72301
48	119245	5280795	8175	442195
49	5320686	6473881	3033866	31553
50	441663	6184	8215	5318891

51	44257675	5281428	8181	197977
52	5281233	11442	31289	5280805
53	5280746	5280899	73114	54611038
54	193876	8181	8914	5281542
55	17473	5283349	454	6442181
56	16211077	6494	68051	46173967
57	5280804	44135670	12314445	11982640
58	3082301	8163	10467	5462438
59	462196	3033866	31284	442664
60	5282102	73114	558173	12305761
61	64982	305	222284	5280804
62	10621	31284	86821	5318880
63	5281643	8215	8163	107905
64	115149	13187	442643	5281807
65	5281673	12367	6184	23915
66	101712	5386591	9548595	5281751
67	441939	73145	5281	5281600
68	128327	92097	5280899	276547
69	5320844	246728	442793	5320844
70	134110	12314445	5281326	168849
71	44257224	10467	5280794	16211710
72	17679	558173	12303924	168114
73	5280441	10976	73145	6475724
74	11948668	6549	92097	10219
75	3085830	67179	6442181	5318893
76	245005	222284	177773	5281753

77	10389806	86821	5386591	117440
78	5383409	101761	13187	44563198
79	5480505	3084282	24502	10455036
80	31553	5281	117301	5481237
81	44258916	9548595	445639	34059
82	72277	31253	5281125	1548943
83	5281777	92785	7605278	181796
84	42607998	5281326	5283349	115149
85	5351212	5280794	12367	64982
86	5281542	12303924	6494	441764
87	44259516	445639	985	73466
88	5281377	5281125	5281794	5280441
89	162464	6442181	246728	6419993
90	6453770	68051	101761	493570
91	5317419	3083613	3084282	168115
92	275182	985	442647	94475
93	5490064	7605278	92785	65064
94	16211710	442643	305	3082301
95	114776	5281794	5997	16211077
96	5317284	5280450	5280450	9954815
97	185617	11005	173183	503737
98	5318880	177773	10976	261859
99	44563159	196359	11005	44563159
100	5281614	442793	1549018	73467
101	92735	5280933	3083613	128327
102	5280459	5280934	6549	6453770

103	49775362	117301	67179	5320686
104	1794427	7794	5282108	98369
105	42608131	5281517	11791	250395
106	5280699	187	5280933	5317419
107	5281807	5997	5280934	442101
108	180948	3893	31253	5281673
109	65084	3520	1548943	44258916
110	97214	73170	5281327	442435
111	94475	5281516	444679	196699
112	276547	173183	3893	5315263
113	3033957	1548943	6433147	358902
114	442195	7765	73170	443421
115	435402	637566	5281517	5281867
116	5281603	643820	7794	189065
117	5318891	1549018	521380	22524410
118	22524411	2969	92138	356660
119	5318893	638011	196359	3085830
120	189065	643779	92885	5281794
121	16142	5281327	5281752	72277
122	381851	444679	173843	366269
123	5281654	11953920	1104	49770696
124	5281775	5282108	5281516	25202038
125	5464155	442647	101616	65033
126	5281675	8158	2969	5383409
127	442662	68346	17100	5318998
128	161671	379	442501	12315119

129	25202038	6433147	187	54600668
130	46173850	8294	637566	86821
131	10219	11791	643820	5281775
132	5320351	10983	7765	5281777
133	503737	173843	8158	440936
134	5280666	8892	62935	9276
135	358902	445070	3083909	65071
136	107971	1549778	5462438	44257224
137	5280633	8038	11142	164146
138	5481237	1104	61416	5282102
139	49770697	92885	638011	5464461
140	44563121	7991	643779	441298
141	12314445	92138	6441405	445070
142	9064	61416	8294	5281793
143	52940094	725	10123	5489486
144	5464461	62935	445070	3033957
145	179651	3083909	379	68051
146	100058	521380	1549778	5281417
147	639665	5318516	643684	11948668
148	5322079	17100	22311	668081
149	93045	442501	440917	5490064
150	5281608	5281752	7462	44563121
151	513197	5281131	68346	37834
152	117440	11142	3520	72616
153	91520	14768	5319022	442810
154	5319744	101616	6441404	91520

155	599030	1118	435402	443322
156	72276	23083748	5318891	2116
157	22524410	643684	11953920	513197
158	9954815	10494	11230	5464155
159	3446460	5282110	8892	442662
160	443354	1123	3081930	11005
161	34059	5319022	23135	162350
162	46173969	7780	8038	9979767
163	9979767	10123	5318893	46173966
164	5481964	23135	6857681	443354
165	480768	5462438	10983	44257675
166	73160	22311	5318880	158940
167	5281789	440917	5282110	46173850
168	44563198	7462	5281867	98570
169	200662	6042	10494	5322079
170	173183	435402	44563198	5281377
171	65071	168928	44563121	107971
172	161388	122097	725	5280933
173	160237	6857681	12304682	161388
174	23915	276547	7991	445639
175	164146	10430	6042	5280699
176	442101	5281565	480768	442514
177	5280343	6441405	168928	100058
178	11982640	5281867	5318516	5280450
179	158940	73299	96946	5280459
180	161036	119	5281565	5280633

181	6850754	6441404	24832090	46173965
182	72323	11230	11083898	363452
183	5280445	3081930	5281131	442217
184	31378	11083898	276547	6758
185	73299	5273621	72625	10389806
186	392443	96946	7780	6441404
187	5318624	5318893	6438142	73114
188	6037	24832090	15694585	72625
189	54611038	5318880	6857559	42607998
190	5280795	12304682	5273621	6850754
191	73466	200146	442217	162464
192	5318999	198016	65033	5839585
193	12303924	247	23083748	599030
194	442793	30247	108053	44260098
195	19009	15694585	9954815	92138
196	5997	442048	200146	16061280
197	99091	5316148	11982640	442793
198	54600668	5318891	13872451	185617
199	5379096	108062	442048	609998
200	162350	5018391	5316148	5318624
201	5481234	6857559	3428129	5281131
202	73467	44563121	1123	68081
203	5281691	480768	14768	5480505
204	167691	44563198	1118	91458
205	197469	637520	44563159	5281125
206	167718	442217	11463	99091

207	188323	65033	10545	44257399
208	3081405	64945	73299	6438142
209	7098673	33510	5317652	5281643
210	12315119	637511	51394521	42608131
211	6419993	108053	519662	156073
212	5317652	9954815	6431456	5320462
213	44260098	6438142	122097	177023
214	441893	5317652	5018391	8193
215	638278	10545	6987	392443
216	46173967	3428129	7439	167718
217	167452	403773	10219	5320351
218	65033	10219	5280951	93045
219	261859	1550828	191055	24197663
220	5281406	72616	196699	52940094
221	88708	98570	353825	5281603
222	119041	51394521	403773	44135670
223	49770696	519662	1550828	14986
224	5281417	6431456	72616	65084
225	12305761	6987	98570	126543
226	5462438	7439	7438	442266
227	167551	5281575	30247	5281792
228	44558930	5280951	5318998	5281654
229	14427376	5318998	5353656	120693
230	356660	5353656	68094	92735
231	444679	68094	521710	5281406
232	17751009	167718	42608147	3081405

233	52948006	11463	5281575	73160
234	99474	72625	10430	107848
235	442217	73401	167718	3084742
236	5489486	191055	10455036	5281608
237	145659	61361	64945	6441405
238	163776	91457	637511	24832101
239	161294	353825	3515	6167
240	5281803	11982640	6651	163776
241	5315263	13872451	10719	5281754
242	13872451	44563159	119	10233
243	119396	161388	91457	12309507
244	9548595	521710	637520	442643
245	91439	42608147	442195	54676854
246	5318869	10212	61361	11442
247	443639	68081	389885	5281675
248	222284	6651	2774722	114776
249	168115		8815	5281855
250	493570			441663
251	3428129			3446460
252	5280863			4970
253	12309507			24752823
254	131900			88708
255	10494			107938
256	442514			18772499
257	5839585			49770697
258	2116			3082205

259	107848	643684
260	3220	5646
261	442810	5318869
262	439435	161036
263	5281612	10154
264	282014	6857681
265	46173968	92885
266	10455036	969516
267	441298	185605
268	363452	7098673
269	185605	7605278
270	181796	1309
271	440936	442647
272	34458	13872451
273	91458	114843
274	101761	10143
275	969516	6442675
276	5318998	5280666
277	114829	6037
278	5281753	282014
279	1548943	576072
280	14986	5281614
281	443322	5281752
282	168928	168136
283	3084282	108053
284	100780	131594

285	6167	3661
286	6442675	519662
287	5281605	6857559
288	5280443	5984
289	5646	101300
290	5281326	16573
291	98570	161871
292	155256	439503
293	64945	22179
294	5281327	124966
295	5320462	229851
296	5281852	34458
297	442435	92265
298	6441405	932
299	72301	117301
300	5281125	131592
301	72310	6760
302	76573	10659
303	5281794	6613
304	5280961	98455
305	5280794	10302
306	5320946	
307	72	
308	2703	
309	14608889	
310	609998	

311	439503
312	5281855
313	160254
314	101301
315	24752823
316	5281628
317	5281858
318	5319322
319	92097
320	643684
321	72704
322	356662
323	161871
324	5281708
325	985
326	5281617
327	68406
328	5280378
329	4501
330	576072
331	689043
332	5281707
333	442977
334	161487
335	10168
336	73145

337	5281752
338	5154
339	22179
340	5321317
341	160876
342	16061280
343	37834
344	73337
345	46173970
346	668081
347	72307
348	16573
349	445858
350	5280373
351	5280442
352	10154
353	12304682
354	442009
355	338
356	92785
357	1309
358	20055073
359	73170
360	8215
361	120693
362	98369

363	5280933
364	6438142
365	5281849
366	10233
367	46173966
368	72322
369	9276
370	11005
371	92885
372	6442181
373	72616
374	443421
375	443720
376	4970
377	4382
378	10207
379	72625
380	86821
381	1104
382	932
383	736186
384	5280450
385	445639
386	5281867
387	5280489
388	370

389	5281232
390	73114
391	443422
392	10467
393	5281
394	318797
395	681
396	10639
397	2353
398	525
399	366269
400	44257206
401	152867
402	54670067
403	160142
404	196699
405	403773
406	54676038
407	159888
408	46173971
409	44559448
410	6758
411	54678501
412	442048
413	194774
414	96946

415	12409
416	107938
417	442266
418	5316148
419	168114
420	5281416
421	6251
422	7605278
423	68051
424	89841
425	161271
426	3893
427	44257399
428	181105
429	6036
430	200146
431	177023
432	10227
433	717531
434	5281754
435	3082205
436	5280934
437	66308
438	8468
439	92265
440	124966

441	10143
442	5984
443	624381
444	68313
445	6441404
446	875
447	5281607
448	442643
449	8742
450	626608
451	101616
452	5280899
453	353825
454	10742
455	65078
456	5281832
457	5793
458	8768
459	5610
460	151010
461	360850
462	5202
463	73401
464	6450278
465	1130
466	3884

467	5281575
468	135
469	13584
470	516811
471	114843
472	2969
473	11442
474	643757
475	10208
476	637542
477	3314
478	26948
479	10983
480	389885
481	8357
482	10922465
483	33510
484	95259
485	171548
486	699414
487	101300
488	108053
489	3806
490	117301
491	229851
492	185071

493	444972
494	6613
495	445070
496	119039
497	3083909
498	442943
499	6439929
500	94253
501	442424
502	526503
503	359
504	7478
505	638024
506	98455
507	11635
508	127567
509	439353
510	379
511	28397
512	46173965
513	5281428
514	23135
515	892
516	5429
517	68079
518	5281426

519	3661
520	6267
521	7431
522	441437
523	164642
524	164676
525	938
526	160600
527	8193
528	6027
529	5281131
530	243
531	114917
532	785
533	8222
534	725
535	105118
536	442647
537	12401
538	61416
539	6431456
540	10659
541	131592
542	1118
543	5353656
544	91466

545	171455
546	114850
547	66548
548	289
549	444539
550	10212
551	1123
552	623940
553	101716
554	5281565
555	131594
556	8181
557	107936
558	5280460
559	68081
560	7420
561	51394521
562	119
563	10364
564	68094
565	6760
566	9548630
567	91457
568	7991
569	15694585
570	126543

571	6137
572	4276
573	167683
574	522266
575	6857559
576	95924
577	8158
578	558173
579	12391
580	115269
581	618688
582	2879
583	92138
584	8914
585	204
586	5281235
587	641668
588	8892
589	5273621
590	442146
591	1110
592	4114
593	91354
594	5032
595	4133
596	5570

597	291264
598	6857681
599	2075
600	127742
601	5281516
602	10332
603	442625
604	2774722
605	92812
606	5318516
607	23205
608	10748
609	68827
610	7780
611	1550828
612	6199
613	165549
614	452191
615	11006
616	10104370
617	637566
618	10302
619	11791
620	10719
621	643820
622	73285

623	5316743
624	3033866
625	72610
626	65188
627	6433147
628	8163
629	31284
630	519662
631	12367
632	571784
633	5317485
634	6989
635	12398
636	6998
637	362908
638	17100
639	442501
640	5386591
641	165536
642	168136
643	12072
644	94174
645	441457
646	5271984
647	6651
648	3515

649	325140
650	5281520
651	3520
652	7361
653	173843
654	6430834
655	11230
656	3885
657	2240
658	5282110
659	5280951
660	247
661	6506548
662	61361
663	442536
664	644020
665	5281517
666	7438
667	14350
668	10658
669	5281515
670	2519
671	554
672	7028
673	521380
674	996

675	6549
676	442938
677	10205
678	169014
679	5283349
680	24832090
681	10430
682	5486616
683	14768
684	6473881
685	244
686	521253
687	120671
688	5280953
689	67179
690	3081930
691	10235
692	774
693	91471
694	7127
695	10976
696	19725
697	5322111
698	177773
699	2355
700	42608147

701	1549778
702	72724
703	27867
704	460
705	101680
706	175002
707	8294
708	443009
709	305
710	5018391
711	19243
712	10955174
713	187
714	107937
715	5282108
716	8175
717	524251
718	234566
719	102667
720	10123
721	191055
722	454
723	64685
724	5319022
725	638011
726	5373727

727	30247
728	61126
729	19244
730	92759
731	13187
732	6494
733	6432469
734	6430313
735	5281404
736	636458
737	660
738	31289
739	8815
740	3083613
741	637520
742	6448
743	7794
744	326
745	1254
746	31244
747	7765
748	6988
749	643779
750	6553876
751	7410
752	261491

753	10545
754	323
755	12160
756	11083898
757	637563
758	68346
759	6987
760	7462
761	329983
762	91195
763	12304613
764	7463
765	637511
766	240
767	1549018
768	8141
769	11463
770	521710
771	24473
772	8082
773	68231
774	7439
775	6427105
776	31253
777	26049
778	159055

779	89594
780	8038
781	2537
782	246728
783	34645
784	9231
785	7929
786	7362
787	440917
788	22311
789	18818
790	66948
791	6654
792	2758
793	11142
794	1201521
795	6184
796	7500
797	14525
798	7809
799	6616
800	14896
801	7302
802	16133932
